# CSF Phosphoproteomics Biomarker Analysis from the Phase 2 Clinical Trial SHINE to Elucidate the Role of CT1812 in Alzheimer’s Disease

**DOI:** 10.1002/alz.095147

**Published:** 2025-01-09

**Authors:** Eunah Cho, Jill Caldwell, Kiran Pandey, Duc M Duong, Nicholas T Seyfried, Anthony O Caggiano, Valentina Di Caro, Mary E. Hamby

**Affiliations:** ^1^ Cognition Therapeutics, Inc, Pittsburgh, PA USA; ^2^ Emtherapro, Atlanta, GA USA; ^3^ Emory University School of Medicine, Atlanta, GA USA; ^4^ Cognition Therapeutics, Purchase, NY USA

## Abstract

**Background:**

The sigma‐2 receptor (S2R) modulator CT1812 is a first‐in‐class investigational therapeutic, currently in Phase 2 clinical trials for Alzheimer’s disease (AD). Preclinical and clinical studies have shown that CT1812 displaces Aβ oligomers from synapses and clears them from the brain into the cerebrospinal fluid, restoring cognitive performance in a transgenic mouse model of AD. To investigate the mechanism of action of CT1812 and enable biomarker discovery, a phosphoproteomic analysis of CSF samples from SHINE‐A was performed.

**Method:**

SHINE is a Phase 2 randomized, double‐blind, placebo‐controlled trial to assess the safety and tolerability of two doses of CT1812 for 6‐months in mild to moderate AD patients (NCT03507790), and an interim analysis was performed on the first 24 patients enrolled (SHINE‐A). Tandem‐mass tag mass spectrometry (TMT‐MS) proteomics and phosphoproteomics were performed on baseline and end‐of‐study CSF samples from SHINE‐A (n = 18) and differential abundance (CT1812 vs placebo; p≤0.05) was assessed. STRING pathway analyses (v 12.0) and comparative analyses between differentially abundant proteins and phosphoproteins were conducted (p≤0.05, p≤0.1). Pearson correlation analyses with altered phosphopeptides and CSF Aβ42 and ADAS‐Cog‐11 were performed. An independent cohort from the SPARC trial assessing the effect of CT1812 in AD patients (NCT03493282) was used for clinical validation of findings.

**Result:**

Altered phosphoproteins identified in SHINE‐A CSF samples included AD‐related proteins, APOE (p≤0.05), CHGB, SPARCL1, and SPP1 (p≤0.1). STRING pathway analysis underlined the role of CT1812 in altering key pathways: “vesicle trafficking”, “regulation of neurofibrillary tangle assembly”, “amyloid fibril formation”, and “low‐density lipoprotein particle receptor catabolic process”, these findings replicated in the SPARC independent cohort. Comparative analyses with proteomic data indicated that 2 phosphoproteins were differentially abundant at the protein level: APOE and MEPE (p≤0.1). Correlation analyses between the phosphoproteome and CSF Aβ42 and ADAS‐Cog‐11 identified highly correlated proteins, APOL1, CHGB, SERPINA10, and TALDO1 (p≤0.05).

**Conclusion:**

The altered phosphorylated proteins and pathways affected by CT1812 highlight a potential impact of CT1812 in modulating amyloid, trafficking, and LDL biology, all pathways dysregulated in AD. Overall, findings support proof of mechanisms of action of CT1812 and the continued clinical development of CT1812.

